# Articular cartilage chondrocytes express aromatase and use enzymes involved in estrogen metabolism

**DOI:** 10.1186/ar4539

**Published:** 2014-04-11

**Authors:** Martin Schicht, Jana Ernst, Andrea Nielitz, Lars Fester, Michael Tsokos, Saskia S Guddat, Lars Bräuer, Judith Bechmann, Karl-Stefan Delank, David Wohlrab, Friedrich Paulsen, Horst Claassen

**Affiliations:** 1 Institute of Anatomy, Department II, Friedrich Alexander University Erlangen-Nϋrnberg, Universitätsstraße 19, 91052 Erlangen, Germany; 2 Institute of Anatomy and Cell Biology, Martin Luther University Halle-Wittenberg, Große Steinstraße 52, 06097 Halle (Saale), Germany; 3 Institute of Neuroanatomy, University Medical Center Hamburg-Eppendorf Martinistraße 52, 20246 Hamburg, Germany; 4 Institute of Legal Medicine and Forensic Sciences, Charité-University Medicine Berlin, Tumstr. 21, 10559 Berlin, Germany; 5 University Hospital of Orthopedics and Physical Medicine, Martin Luther University Halle-Wittenberg, Ernst-Grube-Straße 40, 06120 Halle (Saale), Germany

## Abstract

**Introduction:**

Sex hormones, especially estrogens, have been implicated in articular cartilage metabolism and the pathogenesis of postmenopausal osteoarthritis. The conversion by aromatase (CYP19A1) of androstenedione into estrone (E1) and of testosterone into 17β-estradiol (E2) plays a key role in the endogenous synthesis of estrogens in tissue.

**Methods:**

We analyzed the expression of aromatase (CYP19A1) in immortalized C-28/I2 and T/C-28a2 chondrocytes, as well as in cultured primary human articular chondrocytes and human articular cartilage tissue, by means of RT-PCR, Western blotting and immunohistochemistry. By means of quantitative RT-PCR and enzyme-linked immunosorbent assay, we also determined whether the aromatase inhibitor letrozole influences estrogen metabolism of cultured chondrocytes in immortalized C-28/I2 chondrocytes.

**Results:**

Aromatase mRNA was detected in both immortalized chondrocyte cell lines, in cultured primary human chondrocytes, and in human articular cartilage tissue. By means of Western blot analysis, aromatase was detected at the protein level in articular cartilage taken from various patients of both sexes and different ages. Cultured primary human articular chondrocytes, C-28/I2 and T/C-28a2, and human articular cartilage tissue reacted with antibodies for aromatase. Incubation of C-28/I2 chondrocytes with 10^−11^ M to 10^−7^ M letrozole as an aromatase inhibitor revealed significantly increased amounts of the mRNAs of the enzyme cytochrome P4501A1 (CYP1A1), which is involved in the catagen estrogen metabolism, and of the estrogen receptors ER-α and ER-β. Concomitantly, synthesis of estrone (E1) was significantly downregulated after incubation with letrozole.

**Conclusions:**

We demonstrate that human articular cartilage expresses aromatase at the mRNA and protein levels. Blocking of estrone synthesis by the aromatase inhibitor letrozole is counteracted by an increase in ER-α and ER-β. In addition, CYP1A1, an enzyme involved in catabolic estrogen metabolism, is upregulated. This suggests that articular chondrocytes use ERs functionally. The role of endogenous synthesized estrogens in articular cartilage health remains to be elucidated.

## Introduction

Osteoarthritis (OA) is a multifactorial disease. Current evidence suggests that both mechanical and biochemical factors are involved in its progression [1]. Its incidence is increased in men older than 30 years of age and in women over age 50. It seems likely that women are protected from OA before menopause. Clinical, pathological and epidemiological studies have suggested that women experience OA more often after menopause than before [2] and that hormones, in particular estrogens and androgens, participate in disease outbreak [3-9].

Sex hormone receptors have been discovered on the articular chondrocytes of various species (pig, cattle and human) by using immunohistochemical methods [10]. Cultured primary human articular chondrocytes express estrogen receptors ER-α and ER-β, as well as androgen receptors, at the mRNA and protein levels [11]. However, questions arise regarding whether these ERs are used functionally and whether 17β-estradiol plays a role in articular cartilage metabolism.

**Figure 1:**
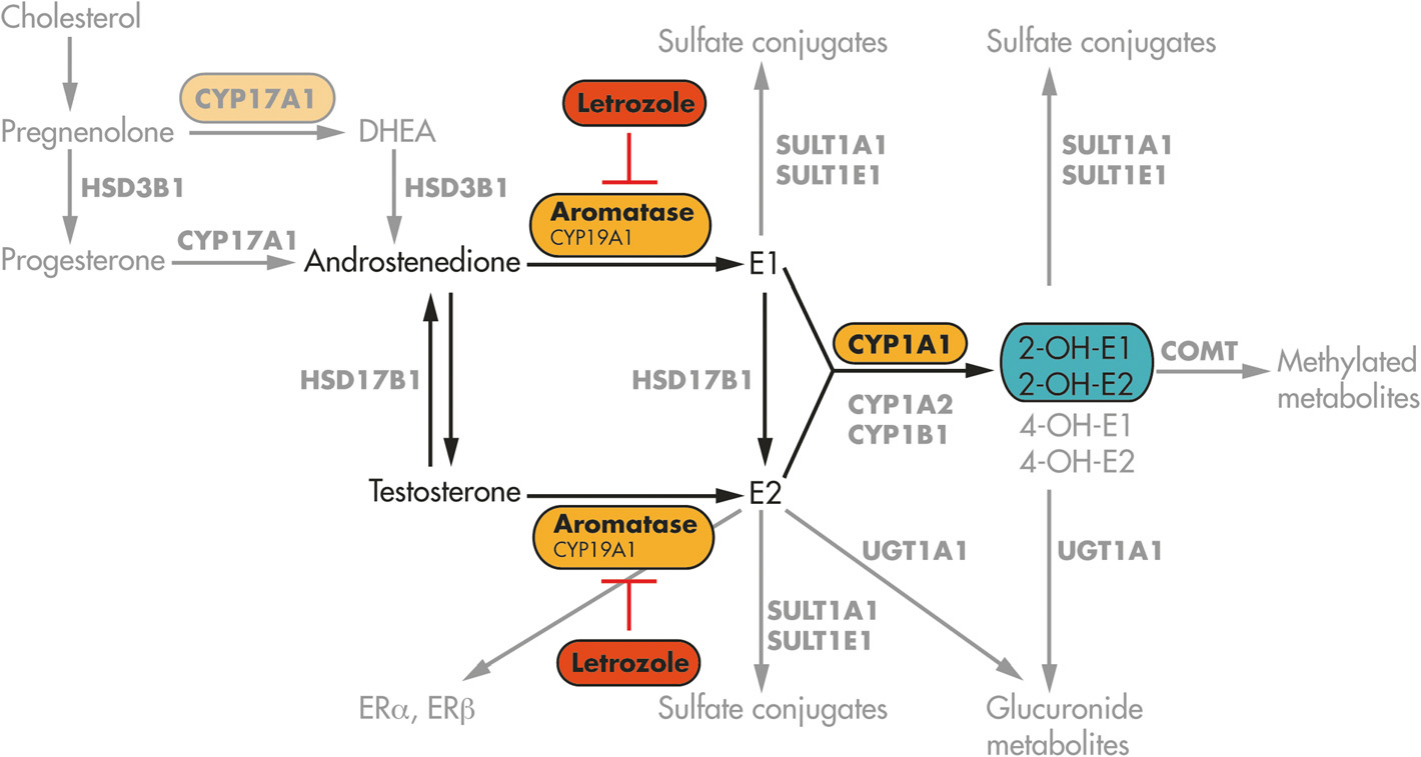
**Schematic showing the details of the estrogen pathway.** Androstenedione and testosterone are converted to estrone (E1) and 17β-estradiol (E2) by aromatase, also named CYP19A1, as the key enzyme of estrogen biosynthesis. Aromatase can be inhibited by letrozole, resulting in blocked synthesis of the estrogens E1 and E2. By contrast, cytochrome P4501A1 (CYP1A1), as an enzyme involved in estrogen catabolism, converts estrone into the hydroxylated catechol estrogen 2-OH-E1. 2-OH-E2: 2-hydroxyestradiol; 4-OH-E1: 4-hydroxyestrone; 4-OH-E2: 4-hydroxyestradiol; COMT: Catechol-*O*-methyltransferase; DHEA: Dehydroepiandrosterone; ER: Estrogen receptor; HSD3B1: hydroxysteroid 3β-dehydrogenase 1. The diagram is modified from a figure published previously by Dumas and Diorio [12].

Aromatase (CYP19A1) is a key enzyme in the synthesis of sex hormones and is involved in the aromatization of androstenedione to form estrone (E1) and of testosterone to form 17β-estradiol (E2) (Figure 1). Estrone itself is transformed into 17β-estradiol by the enzyme hydroxysteroid (17β) dehydrogenase HSD17B1 [12]. Aromatase can be inhibited by letrozole [13]. Researchers have previously shown that chondrocytes in the rib and tibial growth plate, as well as in the temporomandibular joints of male and female rats, express aromatase at the mRNA and protein levels, a process required for the production of 17β-estradiol [14]. Endogenous estrogen synthesis has been detected in temporomandibular joint chondrocytes [15] and in the human cartilage cell line HCS-2/8 [16]. In human articular cartilage, aromatase was first detected by immunohistochemistry [17].

In the present study, we analyzed whether cultured primary human articular chondrocytes and immortalized chondrocytes of the cell lines C-28/I2 and T/C-28a2 (isolated from rib cartilage) express the enzyme aromatase at the mRNA and protein levels. In cultured chondrocytes, we studied whether enzymes involved in estrogen metabolism, such as cytochrome P4501A1 (CYP1A1) are influenced by aromatase inhibition with letrozole, followed by blocked synthesis of E1 and E2 estrogens. A further objective of the study was to investigate the influence of letrozole with regard to the expression of ER-α and ER-β on mRNA levels. A future goal of ours is to analyze the role of endogenous estrogens for articular cartilage health with regard to menopausal OA.

## Methods

### Human tissue

The patients provided their informed consent to participate in the project prior to surgery. The study was approved by the Institutional Review Board of Martin Luther University Halle-Wittenberg and was carried out in accordance with the Declaration of Helsinki.

### Chondrocyte culture

For isolation of primary human articular chondrocytes, articular cartilage was obtained from the posterior area of the medial and lateral femoral condyles of two female patients, ages 70 and 71 years, respectively, who underwent total knee replacement in the Department of Orthopedics at Martin Luther University Halle-Wittenberg. To prepare chondrocytes for cell cultures, full-thickness cartilage taken from patient biopsies was used. The available amount of primary chondrocytes was too restricted to perform extensive cell culture experiments, so we chose to examine the chondrocyte cell lines C-28/I2 and T/C-28a2. The immortalized chondrocyte cell lines C-28/I2 and T/C-28a2, which originated from cells isolated from rib cartilage of a 15-year-old female, were transduced with simian virus 40 containing the large T antigen [18]. Both cell lines express *SOX9* as the master gene of chondrocytic cell differentiation. The major difficulty with studying C-28/I2 and T/C-28a2 chondrocytes is that they mainly proliferate and show less expression of genes of matrix synthesis and turnover [19,20]. However, C-28/I2 chondrocytes express significantly higher levels of matrix-degrading proteases compared with T/C-28a2 chondrocytes. The chondrocyte culture was performed as described previously [11].

### Incubation with letrozole

At the end of culture with serum, C-28/I2 and T/C-28a2 chondrocytes were changed to serum-free medium for 2 hours, cells were incubated alone or with a range of concentrations (10^−11^ M, 10^−9^ M and 10^−7^ M) of letrozole (Femara; Novartis Oncology, Nuremberg, Germany) during the two serum-free days (days 6 and 7 or days 8 and 9, respectively). For this purpose, stock solutions of 10^−1^ M letrozole (EtOH-soluble) were prepared and dissolved stepwise with distilled H_2_O (EMD Millipore, Billerica, MA, USA). All experiments were performed in triplicate, and controls were incubated with water instead of the aromatase inhibitor letrozole.

### Human tissues

Articular cartilage samples were obtained from autopsy cases (9 males: ages 22, 24, 28, 33, 41, 48, 59, 66 and 73 years; 7 females: ages 3, 7, 38, 52, 62, 83 and 90 years) from the Institute of Legal Medicine, Charité-University Medicine Berlin, Germany. Samples used for positive controls, such as placenta, testicle, ovary, lung, lacrimal gland, heart, uterus, prostate and nasal turbinate epithelium, were obtained from cadavers donated to the Department of Anatomy and Cell Biology, Martin Luther University Halle-Wittenberg, Germany.

### Reverse transcription

Total RNA from cultured C-28/I2 and T/C-28a2 chondrocytes, from primary human articular chondrocytes and from human ovary were isolated as described previously [11].

### Quantitative RT-PCR

Samples from cultured C-28/I2 chondrocytes were analyzed using the StepOnePlus Real-Time PCR System (Applied Biosystems, Foster City, CA, USA). Real-time RT-PCR was performed with specific primers (Table 1) to allow calculation of the relative abundance of transcripts. The reactions were performed using 10 μl of SYBR Green Master Mix (MESA BLUE qPCR MasterMix Plus for SYBR Assay; Eurogentec, Cologne, Germany) and 3 μl of cDNA. The cycle parameters were 5 minutes at 95°C and 40 three-step cycles of 10 seconds at 95°C, 15 seconds at 60°C and 20 seconds 72°C. Standard curves were generated for each gene using a plasmid dilution series containing the target sequences. All quantitative RT-PCRs (qRT-PCRs) were performed in triplicate, and the changes in gene expression were calculated by the 2-^ΔΔCt^ relative quantitation method. Analysis of the data yielded values relative to the mRNA concentration.

**Table 1:** **Sequences of the primers used for RT-PCR and real-time analysis**^**a**^

**Primer**	**Sense primers (5′ → 3′)**	**Antisense primers (5′ → 3′)**	**Base pair**	**Temperature**
Aromatase	CGA GAT CGA AAT TCT GGT GGA AAA G	TGC AAA ATC CAT ACA GTC TT	177	60°C
CYP1A1	CCT CTT TGG AGC TGG GTT TG	GCT GTG GGG GAT GGT GAA	229	60°C
ERα	CAA TGA CTA TGC TTC AGG CTAC	CCA CCT TTC ATC ATT CCC AC	198	60°C
ERβ	GAG TCC CTG GTG TGA AGC AA	TGA GCA TCC CTC TTT GAA CC	199	60°C
β-actin	CAA GAG ATG GCC ACG GCT GCT	TCC TTC TGC ATC CTG TCG GCA	275	60°C

^a^CYP1A1: Cytochrome P4501A1; ER: Estrogen receptor.

### Protein preparation

Protein was isolated as described previously [11].

### Western blot analysis

For each articular cartilage sample and each positive control sample (see Human tissue), 20 μg protein/lane were prepared under conditions as described previously [11] and incubated with specific antibodies for aromatase (Acris, SM2222P) at 4°C overnight.

### Immunohistology and immunocytochemistry with peroxidase

Human articular cartilage from 28 and 83-year-old male donors were dehydrated and embedded in paraffin. Sections of 3-5 μm were deparaffinized and stained with specific antibody for aromatase (SM2222P; Acris Antibodies, Herford, Germany) at 4°C overnight. The protocol followed was described previously [11].

### Immunocytochemistry with fluorescein isothiocyanate

Immortalized chondrocytes of the cell lines C-28/I2 and T/C-28a2 or primary human articular chondrocytes cultured on glass coverslips were prepared under conditions described previously [11] and incubated with specific antibodies for aromatase (SM2222P; Acris Antibodies) at 4°C overnight. Immunoreactions were analyzed using a fluorescence microscope (BZ-8100; Keyence Deutschland GmbH, Frankfurt, Germany). Double-staining was performed based on the same principle. The second antibody used was goat anti-rabbit calreticulin (ABR-01176; Dianova, Hamburg, Germany) as a marker of endoplasmic reticulum.

### Enzyme-linked immunosorbent assay

A commercially available enzyme-linked immunosorbent assay (ELISA) kit (DRG Instruments, Marburg, Germany) was used manufacturer instruction to quantify the amount of estrone (EIA-4174) in supernatants from stimulated C-28/I2 cells. The analysis was performed using a microplate spectrophotometer (ELISA reader) to measure absorbance (450 nm). The concentrations of the samples are expressed in picograms per milliliter.

### Statistics

The data are expressed as the mean ± standard error of the mean (SEM) of tested samples. Statistical analysis was performed using the Mann-Whitney *U* test or analysis of variance after assessing the normal distribution of the data using InStat statistical software (GraphPad Software, San Diego, CA, USA). *P*-values ≤0.05 were considered statistically significant.

## Results

### Expression of aromatase at mRNA level

**Figure 2:**
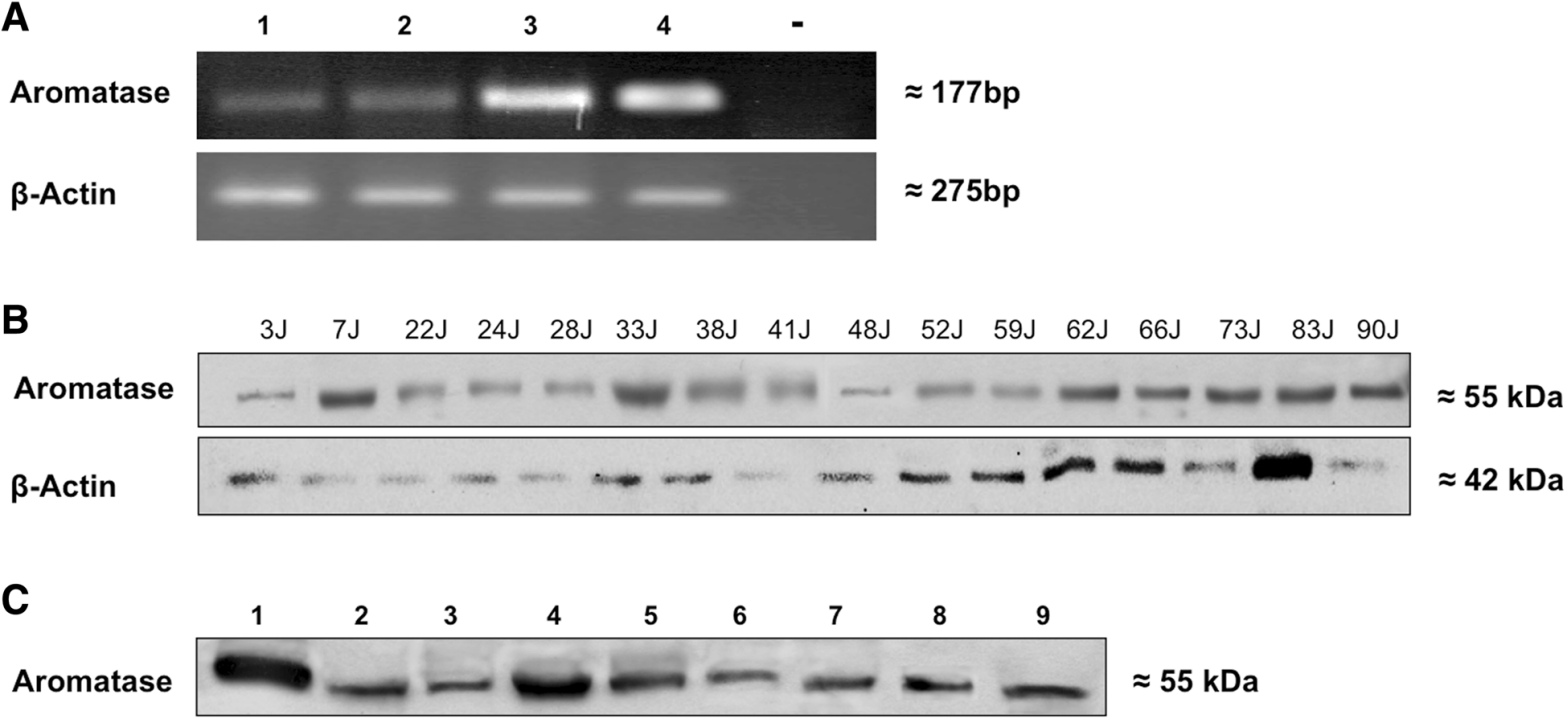
**Detection of aromatase in human articular cartilage tissue, in primary human articular chondrocytes and in chondrocyte cell lines C-28/I2 and T/C-28a2. (A)** Aromatase mRNA (177 bp) was detected in articular cartilage tissue (lane 1), in cultured articular chondrocytes (lane 2) and in immortalized chondrocytes of cell lines C-28/I2 (lane 3) and T/C-28a2 (lane 4). Diethylpyrocarbonate water served as a negative control (lane 5). **(B)** Western blot analysis of aromatase using the antibody SM2222P in articular cartilage tissue from patients of both sexes and different ages. Aromatase protein is detected at 55 kDa in articular cartilage from females ages 3, 7, 38, 52, 62 and 83 years and from men at ages 22, 24, 28, 33, 41, 48, 59, 66 and 73 years. **(C)** Western blot analysis of aromatase in various control tissues. Aromatase protein is found in both primary sex hormone–dependent organs, such as placenta (lane 1), testicle (lane 2), ovary (lane 3), uterus (lane 7) and prostate (lane 8), and in primary non-sex-hormone–dependent organs, such as lung (lane 4), lacrimal gland (lane 5), heart (lane 6) and nasal turbinate epithelium (lane 9).

With specific primers for human aromatase (CYP19A1), a PCR product of the expected size (177 bp) was found in all samples of cultured primary human articular chondrocytes, cultured C-28/I2 and T/C-28a2 chondrocytes, as well as in samples of human articular cartilage tissue (Figure 2A). Sequencing of the bands showed 100% agreement with the corresponding human DNA sequence.

### Expression of aromatase at protein level

Western blot analysis for aromatase revealed that articular cartilage in all samples taken from both sexes and different ages expressed the enzymes at the expected molecular weight (55 kDa) (Figure 2B). The expression pattern of the enzymes, in comparison with β-actin, did not depend on sex or age. Both sexes showed stronger expression of the enzyme aromatase, beginning in the seventh decade of life, compared with younger participants. Furthermore, aromatase was detected in placenta, testis, ovary, uterus, prostate and in primary non-sex-hormone–dependent organs such as lung, lacrimal gland, heart and nasal turbinate epithelium (Figure 2C).

### Immunocytochemistry and immunohistochemistry

**Figure 3:**
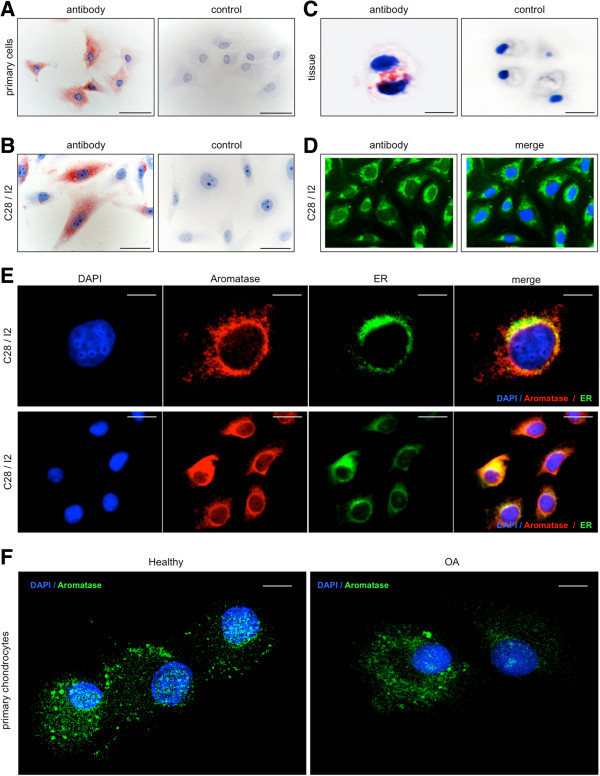
**Immunocytochemical and immunohistochemical staining of aromatase or aromatase combined with calreticulin in human articular cartilage tissue, in primary human articular chondrocytes and in immortalized chondrocytes of cell line C-28/I2. (A)** Cultured primary articular chondrocytes react with aromatase antibody as revealed by red staining. **(B)** Cultured chondrocytes of cell line C-28/I2 react with the aromatase antibody. Control cells do not react. **(C)** Articular cartilage tissue shows immunoreactivity for aromatase, and the control remains unstained. **(D)** Localization of aromatase by immunofluorescence. The slides present staining of nuclei by 4′,6-diamidino-2-phenylindole (DAPI) and of cytoplasm with fluorescein isothiocyanate (FITC)–conjugated antibodies to aromatase (antibody) or combined staining of nuclei and aromatase (merged image). Cytoplasm of cultured C-28/I2 cells show positive reaction for aromatase. **(E)** Localization of aromatase combined with calreticulin by immunofluorescence. The slides present staining of nuclei by DAPI; staining of cytoplasm with FITC-conjugated secondary antibodies against aromatase or calreticulin; and combined staining of nuclei, aromatase and calreticulin (merged image). As shown by single-cell laser-scanning microscopy, staining for aromatase and calreticulin is observed at approximately identical cytoplasmic sites. ER: Endoplasmic reticulum. **(F)** Primary human articular chondrocytes from knee joint tissue with and without osteoarthritis (OA) show aromatase immunostaining equally in all chondrocytes. Scale bars: 50 μm **(A and B)**, 10 μm **(C and E)** and 20μm **(F)**.

The cytoplasm of cultured primary human articular chondrocytes and cultured, immortalized C-28/I2 chondrocytes were immunostained by a specific antibody for aromatase (Figures 3A, 3B and 3D). Cells of the immortalized chondrocyte cell line T/C-28a2 showed an identical staining pattern (not shown). Additionally, chondrocytes of all articular cartilage samples reacted with the antibody to aromatase (Figure 3C). However, a zone-dependent expression of this enzyme was not observed. Exact localization of the enzyme by immunocytochemical staining of nuclei by 4′,6-diamidino-2-phenylindole and the aromatase reactivity by antibody SM2222P were analyzed using laser scanning microscopy (Figure 3E). Positive reactivity was visible only as a small rim of cytoplasm around the nucleus of immortalized chondrocytes C-28/I2 and T/C-28a2 with antibodies for aromatase. Aromatase showed expression on endoplasmic reticulum. Double staining with antibodies for aromatase and calreticulin as markers for endoplasmic reticulum revealed partial overlap of the staining patterns for both proteins (Figure 3E). In primary human articular cartilage chondrocytes from tissue with and without OA aromatase is equally expressed in all cells (Figure 3F).

### Letrozole incubation of C-28/I2 chondrocytes

**Figure 4:**
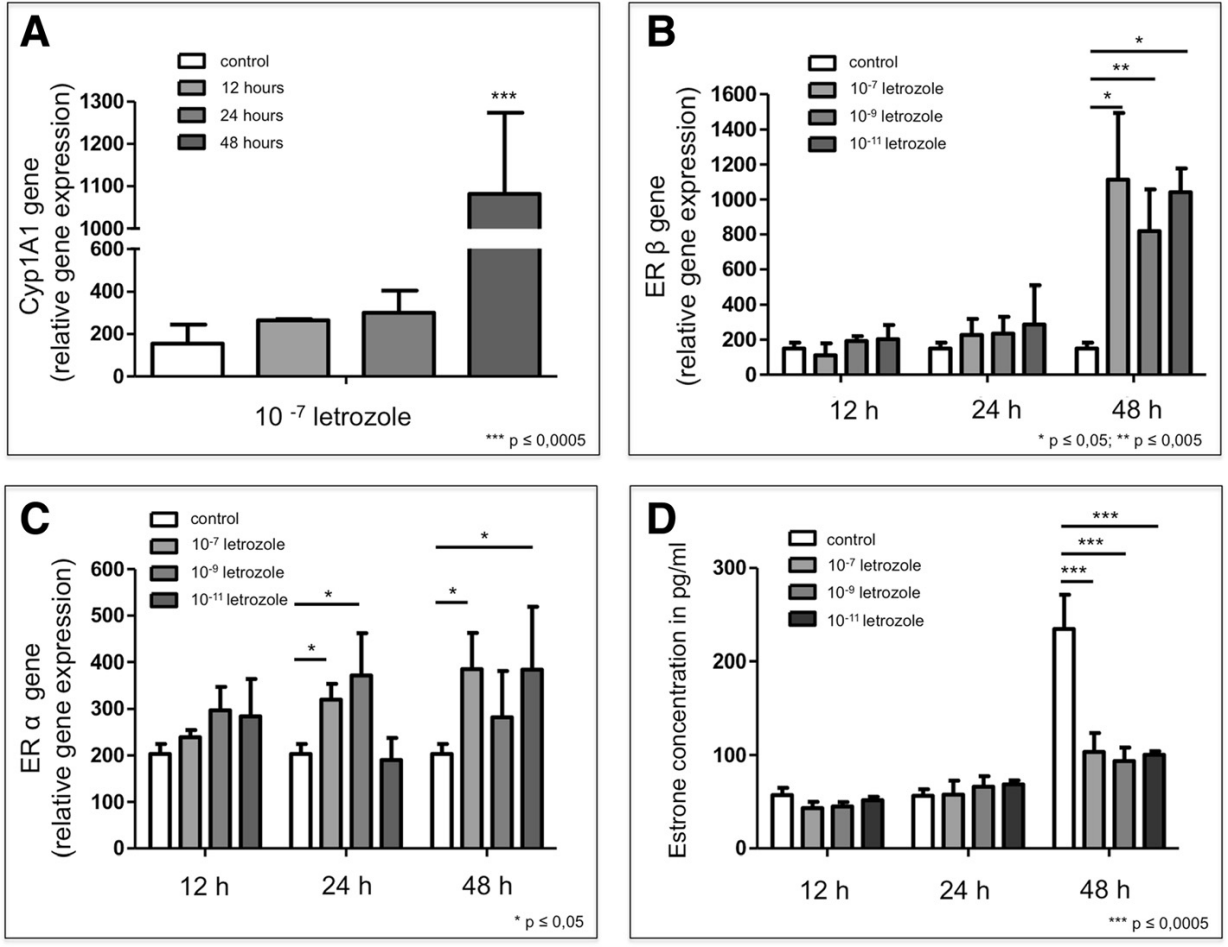
**Influence of letrozole on mRNA concentrations of cytochrome P4501A1 and estrogen receptors ER-α and ER-β in cultured C-28/I2 chondrocytes analyzed by quantitative RT-PCR and on estrone concentration of these cells assessed by enzyme-linked immunosorbent assay. (A)** Compared to controls, chondrocytes cultured without aromatase inhibitor incubated with 10^−11^ M to 10^−7^ M letrozole show significantly increased expression of cytochrome P4501A1 (CYP1A1) mRNA after 48 hours with 10^−7^ M letrozole. We also observed a tendency toward this effect after incubation for 12 and 24 hours using concentrations of 10^−9^ M and 10^−7^ M letrozole. **(B)** Quantitative RT-PCR (qRT-PCR) shows significantly increased expression of estrogen receptor ER-β mRNA following incubation with 10^−11^ M to 10^−7^ M letrozole for 48 hours. No regulatory influence was observed after incubation with 10^−11^ M to 10^−7^ M letrozole for 12 and 24 hours. **(C)** qRT-PCR revealed significantly increased expression of ER-α mRNA following incubation with 10^−7^ M and 10^−11^ M letrozole for 24 and 48 hours. **(D)** In comparison to control chondrocytes cultured without aromatase inhibitor, incubation with 10^−11^ M to 10^−7^ M letrozole for 48 hours led to significantly downregulated expression of estrone (E1) protein as revealed by enzyme-linked immunosorbent assay. All experiments were performed in triplicates (*n* = 3).

To test the influence of the aromatase inhibitor letrozole on the expression of the catabolic enzyme cytochrome P4501A1 (CYP1A1) and ER-α and ER-β, C-28/I2 chondrocytes were incubated with different concentrations of 10^−11^ M - 10^−7^ M letrozole for 12, 24 and 48 hours. qRT-PCR analysis revealed statistically significant increased mRNA expression of CYP1A1 after incubation with 10^−7^ M letrozole for 48 hours compared to control (Figure 4A), whereas, after 12 and 24 hours, no increased expression of CYP1A mRNA could be detected. Also, incubation with 10^−11^ M - 10^−9^ M letrozole showed no effect on CYP1A1 mRNA expression. ER-β mRNA was significantly increased after 48-hour incubation with 10^−11^ M to 10^−7^ M letrozole (Figure 4B), whereas, after 12 and 24 hours of incubation, no effect was observed. Furthermore, ER-α mRNA was significantly increased after incubation with 10^−11^ M to 10^−7^ M letrozole for 24 and 48 hours (Figure 4C). Incubation for 12 hours showed no significant influence on the expression of ER-α mRNA. The concentration of estrone (E1) in articular chondrocytes was quantified by means of ELISA. In comparison to controls, a statistically significant downregulation of this hormone was observed after incubation with 10^−11^ M to 10^−7^ M letrozole for 48 hours (Figure 4D). Incubations for 12 or 24 hours showed no effect on the concentration of estrone.

## Discussion

High estradiol levels in late puberty induce growth plate closure and thus cessation of growth in humans [21]. The enzyme aromatase (CYP19A1), which converts androstenedione into 17β-estradiol, is expressed in the hypertrophic zone of human growth plates [22]. These facts are consistent with a role for local estrogen production in the paracrine control of long-bone growth. On the basis of studies of patients with a mutated aromatase gene or defective ER-α, it has become clear that the action of estrogen is indispensable for normal pubertal growth [23,24]. Inhibition of estrogen action by aromatase inhibitors such as letrozole seems to decelerate the process of growth plate fusion [13]. Therefore, letrozole can be used therapeutically to increase predicted adult height in boys with idiopathic short stature [25]. Compared to controls, the height of the epiphyseal growth plate is increased by 12% in letrozole-treated mice [26].

Locally synthesized 17β-estradiol is useful for growth plate chondrocytes of the cell line HCS-2/8 because it stimulates proliferation and protects against spontaneous apoptosis [16]. On the basis of the clinical observation that women suffer more from OA with the beginning of menopause [1], it can be asked whether estrogens play a role in this disease and, in particular, if endogenously produced estrogens are protective of articular cartilage. In postmenopausal women, estradiol is produced at extragonadal sites such as in cells of adipose tissue and in numerous sites of the brain acting as paracrine factors [27,28]. Articular chondrocytes of rats are capable of synthesizing sex steroid hormones locally from dehydroepiandrosterone (DHEA) [29].

In our present study, we detected aromatase in cultured primary human articular chondrocytes and in immortalized chondrocytes of cell lines C-28/I2 and T/C-28a2 at the mRNA and protein levels. Both cell lines originated from chondrocytes isolated from the rib cartilage of a 15-year-old female [18]. Because ERs were found in cell lines C-28/I2 and T/C-28a2 originating from rib cartilage [11], it is not surprising that both chondrocytes of rib cartilage and chondrocytes of articular cartilage express the enzyme aromatase. In relation to the housekeeping gene β-actin, at the mRNA level, aromatase is expressed more strongly in the immortalized cell lines C-28/I2 and T/C-28a2 compared to articular cartilage tissue and cultured primary articular chondrocytes (Figure 2A). However, immortalized chondrocytes from rib cartilage may differ from primary articular chondrocytes with regard to the strength of protein expression [30]. As stated by Finger and colleagues [19], the expression of *SOX9* as a marker gene of chondrocyte differentiation is at a significant level in both cell lines, whereas extracellular matrix proteins and matrix-degrading proteases are rarely expressed. In addition, it must be taken into account that primary articular chondrocytes were derived from endoprosthesis surgery in two women, ages 70 and 71 years, respectively, who had OA of the knee (see the Methods section). Because aromatase supplies articular cartilage, with endogenous estrogens hypothesized to be cartilage-protective, the weaker expression of this enzyme may be induced by OA. At the protein level, the observed expression of aromatase is not sex-specific when age-matched females and males are compared (Figure 2B). Compared to females and males ages 2 to 59 years, aromatase expression in both sexes is stronger beginning at 60 years of age. Perhaps the stronger expression of aromatase as an enzyme-converting androstenedione in estrogens reflects a lack of endogenous estrogens in the articular cartilage of women and men at advanced ages. In human bone tissue, the amount of aromatase mRNA expression was found to correlate positively with the degree of osteoporotic changes [17]. Taken together, analogously to bone tissue, endogenous estrogens might be cartilage-protective for both sexes at advanced ages. Unfortunately, this hypothesis does not explain the clinical observation that women are generally protected against OA until menopause, but men can be affected beginning in approximately the fourth decade of life.

Using immunohistochemistry, we localized the enzyme in the cytoplasm of the cell lines studied. Additionally, a predominant localization of aromatase near the endoplasmic reticulum was shown by double-staining with antibodies to aromatase and calreticulin. Aromatase was previously found to be undetectable in rabbit articular cartilage [31]. However, Bellino detected another enzyme specific for estrogen metabolism: 17β-hydroxysteroid dehydrogenase. Because aromatase is produced in human articular chondrocytes, we speculate that these cells can synthesize 17β-estradiol and that endogenous estrogens play a role in articular cartilage health. This hypothesis is confirmed by experiments with DHEA-treated chondrocytes where the aromatase inhibitor letrozole locally reduced estrogen synthesis followed by increased expression of matrix metalloproteinase 13 and decreased synthesis of type II collagen [32]. Both effects are damaging to the articular cartilage matrix. Furthermore, letrozole used for treatment of early postmenopausal breast cancer has been reported to induce arthralgia [33]. By contrast, it has been speculated that aromatase plays an important role in the development of temporomandibular joint disorders by producing estrogens [14].

As for OA, endogenous estradiol is assumed to play a role in bone health and has been discussed with regard to its involvement in bone fractures [34]. A novel mutation of the human aromatase gene was shown to be involved in bone growth and mineralization [35]. Estrogen deprivation was established as a central mechanism in the development of osteoporosis with aging [36]. In postmenopausal women on dialysis, who have a high risk for developing mineral and bone disorders, it was shown that endogenous estrogens may prevent bone loss [37].

Furthermore, we asked what happens in articular cartilage metabolism when estrogen synthesis is blocked by the aromatase inhibitor letrozole (Figure 1A). Following incubation of C-28/I2 chondrocytes with 10^−11^ M to 10^−7^ M letrozole for 48 hours, CYP1A1 mRNA was significantly increased compared to controls. The enzyme CYP1A1 participates in estrogen catabolism and causes 2-hydroxylation of estrone (E1) [12,38]. Concomitantly, mRNA expression of ER-α and ER-β was significantly increased. We hypothesize that the chondrocytes tend to utilize the remaining parts of estrogens in their vicinity, thereby increasing the amounts of their ERs. The accumulation of the catabolic enzyme CYP1A1 may be interpreted as an attempt by the chondrocytes to hydroxylate existing remnants of estrone to catechol estrogens.

Serum concentrations of free and total estradiol, as well as of estrogen metabolites such as 2-hydroxyestrone (2-OH-E1), 2-hydroxyestradiol (2-OH-E2) and 16α-hydroxyestrone, have been shown to be involved in female OA [39] (Figure 1). Gao and colleagues [39] showed that free and total estradiol are reduced, but the total 2-OH-E2 level was increased, in postmenopausal women. In addition, it should be noted that total 2-OH-E1 concentration is positively correlated with the total estradiol level in premenopausal women with OA. Afzal and Khanam [40] observed high levels of the proinflammatory cytokine interleukin 6 accompanied by decreased estrogen levels in postmenopausal female OA patients. From another point of view, it was stated that estrogens reduce cellular aging in chondrocytes by deceleration of telomere attrition [41].

Taken together, articular chondrocytes of both sexes possess the enzyme aromatase, also named CYP19A1, at the mRNA and protein levels. Chondrocytes tend to increase their amounts of ER-α and ER-β when estrone synthesis is blocked by the aromatase inhibitor letrozole. Furthermore, the catabolic enzyme CYP1A1, which hydroxylates estrone, is increased after blocking of estrone synthesis with letrozole.

## Conclusion

We conclude that chondrocytes react with a change in the expression of their ERs following intervention in the anabolic pathway of estrogen synthesis (Figure 1). The results presented herein reveal that human articular cartilage expresses the enzyme aromatase (in mRNA and protein). The results also suggest that articular chondrocytes are able to produce estrogens by themselves and are independent of external estrogen metabolism. Furthermore, articular chondrocytes use ER-α and ER-β functionally. The influence of estrogens on cartilage metabolism has been shown in several recent studies, which reflects the complexity of negative and positive effects of sex hormones on articular cartilage metabolism. Further investigations are needed to elucidate the special pathways and functions of estrogens, especially endogenous synthesized estrogens, in articular cartilage metabolism with regard to menopausal OA.

## Abbreviations

CYP19A1: Aromatase; CYP1A1: Cytochrome P4501A1; E1: Estrone; ER: Estrogen receptor; OA: Osteoarthritis; RA: Rheumatoid arthritis.

## Competing interests

The authors declare that they have no competing interests.

## Authors’ contributions

All authors were involved in drafting the manuscript or revising it critically for important intellectual content. MS, JE, AN and JB performed the research. LF, MT, SG, KSD and DW provided numerous samples for experiments, performed endoprosthetic surgery and selected articular cartilage used for chondrocyte cultures. MS, FP, LB and HC participated in the study conception and design as well as the analysis and interpretation of data. All authors read and approved the final manuscript.
